# Effects of Modest Hypoxia and Exercise on Cardiac Function, Sleep-Activity, Negative Geotaxis Behavior of Aged Female *Drosophila*

**DOI:** 10.3389/fphys.2019.01610

**Published:** 2020-01-21

**Authors:** Qiu Fang Li, Hui Wang, Lan Zheng, Fan Yang, Han Zhe Li, Jin Xiu Li, Dan Cheng, Kai Lu, Yang Liu

**Affiliations:** Key Laboratory of Physical Fitness and Exercise Rehabilitation of Hunan Province, Hunan Normal University, Changsha, China

**Keywords:** normobaric hypoxia, exercise training, cardiac, sleep-activity, negative geotaxis, aged *Drosophila*

## Abstract

Mild normobaric hypoxia (NH) and modest exercise have multiple beneficial effects on health, but the changes in physiological function induced by NH and/or exercise remain unclear. The purpose of this investigation was to examine the specific effects of NH and/or exercise on cardiac function and myocardial structure and behavior including sleep-activity and negative geotaxis in aged *Drosophila.* We also assessed the survival rate of flies after hypoxia and/or exercise. One-thousand wild-type w^1118^ virgin female flies were randomly divided into four groups and treated with NH and/or exercise from ages 3–6 weeks. We found that exercise remarkably delayed the decline of actin and myosin and the age-related changes in cardiac structure, improved abnormal cardiac contraction, and enhanced the cardiac pumping force by inducing cardiac hypertrophy and delaying deterioration of cardiac contractility and diastolic compliance, and improved abnormal heart contraction. NH also increased the content of actin and myosin, but induced a decrease in heart diameter and heart rate, as well as an increase in the number of mitochondria and deeper sleep, which may be the manifestation of energy saving under long-term hypoxia. Both NH and exercise improved sleep quality and climbing ability of aged flies, as well as extended the maximum life span, which shows the benefits of hypoxia and exercise. Finally, the superposition of NH and exercise did not impart any obvious physiological and behavior improvement. Therefore, it is necessary to further explore the appropriate combination of hypoxia and exercise.

## Introduction

A hallmark feature of aging is the progressive decline in physiological structure and function accompanied by a steady increase in mortality caused by a range of age-related diseases (Jones and Grotewiel, 2011; He and Jasper, 2014). Of all the non-communicable diseases, cardiovascular diseases pervade the world’s aging population at all socioeconomic classes (Lozano et al., 2012). Age-related behavioral changes in humans, including progressive decline in locomotor ability and circadian rhythmicity such as dyskinesia and loss of sleep consolidation, have also been receiving attention (Duffy and Czeisler, 2002; Bliwise et al., 2009; Jones and Grotewiel, 2011; Duffy et al., 2015; Lanza et al., 2019). The symptoms of aging are currently developing at a younger age, and informal self-reporting in most individuals beyond the age of ∼30–35 years has revealed a decline in age-related function (Jones and Grotewiel, 2011). Improved treatment of age-related physiological and behavioral degradation may therefore improve the quality of life of the elderly. Despite significant advances in modern medical techniques, the prevention of disease remains more economical and effective than treatment.

Different physical exercise protocols can lead to beneficial effects or pathological outcomes. Similarly, different physiological stresses of hypoxia also generate two diametrically opposite results (Messmer-Blust et al., 2009). Relative milder hypoxic exposure and modest exercise training can enhance both physical and mental capacities, resulting in increased aerobic performance capacity (Fulco et al., 2000; Kavaliauskas et al., 2017). Evidence demonstrated that mild hypoxic preconditioning and hypoxia-mimetic agents are beneficial to cerebral ischemic stroke and some neurodegenerative disorders. And hypoxia-inducible factor 1 may be a viable target for preventing or ameliorating pathophysiological conditions, such as neurodegenerative diseases (Correia and Moreira, 2010). In addition, exercise training is now a well-established preventive measure and therapy for cardiovascular diseases (Villella and Villella, 2014; Cattadori et al., 2018). Also, as an important factor affecting the quality of life of the elderly, autonomous activity and sleep status are highly correlated with happiness and mental state (Farholm et al., 2017), while autonomous physical activity and sleep quality can also be improved through exercise (Edinger et al., 1993; Wang and Youngstedt, 2014; Bonardi et al., 2016). However, studies have also shown that hypoxia, which is equivalent to conditions of mild to moderate altitudes, can lead to improved and deteriorated sleep (Berssenbrugge et al., 1984; Kinsman et al., 2005; Sargent et al., 2013). Thus, the effects of acclimatization on sleep quality under hypoxia requires further investigation.

*Drosophila melanogaster* is regarded as an excellent model for studying aging (Nishimura et al., 2011), cardiac function (Ocorr et al., 2014), circadian rhythms, and sleep parameters (Andretic and Shaw, 2005; Chiu et al., 2010). The linear heart, which pumps hemolymph, is situated in the dorsal midline of adult *Drosophila* (Shah et al., 2011). Studies have shown that during *Drosophila* metamorphosis, the heart undergoes remodeling, which ultimately results in a formation of the adult heart that remains unchanged until death (Schnorrer and Dickson, 2004; Augustin and Partridge, 2009; Lehmacher et al., 2012). Similar to vertebrates, fly muscles undergo characteristic changes during aging, including Z-disk disruption, reduction in the expression of sarcomere components, disruption of thin filament organization, and reduced flight and climbing ability (Miller et al., 2008; Perkins and Tanentzapf, 2014). Furthermore, various age-related behavioral changes in humans have also been observed in *Drosophila*, including locomotor impairment (Grotewiel et al., 2005) and poor sleep quality (Koh et al., 2006; Vienne et al., 2016). Based on the risk and complexity of applying exercise and normobaric hypoxia (NH) protocols to older adults, as well as the relatively shorter experimental period, easy breeding and rearing, simple and feasible detection methods, and age-related diseases also observed in human beings, *Drosophila* has become an excellent model for studying aging.

Mild NH and modest exercise training can instill beneficial effects on the body. Our research team has explored optimal exercise and NH protocols (Zheng et al., 2015; Wang et al., 2017). The purpose of the present investigation was to study changes in cardiac structure and function and behaviors that include sleep-activity and negative geotaxis of aged flies induced by NH and exercise. We also assessed the survival rate of flies after hypoxia and/or exercise. In addition, this study also explored the effects of a combination of hypoxic exposure strategies and exercise patterns.

## Materials and Methods

### *Drosophila* Stocks, Diet and Rearing

Wild-type w^1118^ virgin female flies were collected within 8 h after eclosion and reared on a yeast-maltose-cornmeal-sucrose-agar diet with propionic acid and Tegosept to limit bacterial growth. All 20 flies were housed in a vial capped with a sponge and containing diet on the bottom, and these were raised in standard environmental conditions (25°C, 50% relative humidity, 12-h light/dark cycle). The flies were transferred to clean vials containing fresh food every 2 days without anesthesia.

### NH and Exercise Training Devices and Protocols

The flies were randomly divided into four groups: normoxic control group (NC), normoxic exercise group (NE), hypoxic control group (HC), and hypoxia-exercise group (HE). All experiments were conducted in a normobaric environment. The hypoxia chamber was created using a 600-mL glass container and two plastic tubes. Two holes were drilled into the container, and plastic tubes were inserted into each hole, to which screw caps and a rubber band were attached. A mixture of O_2_ and N_2_ gas was passed through one tube into the container and bubbled through distilled water to maintain the humidity of the container, and then the air flowed outward from the other tube that was immersed in water to avoid the intake of air from the outside. O_2_ content within the container was monitored constantly using a ToxiRAE II oxygen monitor (RAE Systems, Sunnyvale, CA, United States). We took advantage of the flies’ natural negative geotaxis behavior to construct the exercise device to induce *Drosophila* continuous upward walking. Vials with diet housing 20 flies each were loaded horizontally into a steel tube that was rotated along its horizontal axis by an electric motor with a gear regulating its shaft speed. Thus, with the accompanying rotating steel tube, each vial was rotated along its long axis, which stimulated the flies to climb. Most flies continued to respond by climbing throughout the exercise period. The few that failed to climb were actively walking on the inner wall of the vial. Based on the best exercise program that has been determined, we selected continuous exercise for 2.5 h (Zheng et al., 2015).

Studies show that flies can live a normal life span at 4% O_2_, but they cannot reproduce and display lasting cardiac dysfunction (Zarndt et al., 2015). By contrast, the survival rate of F1 embryos of the parental flies under with 6% O_2_ was slightly reduced, but no significant effect on the body size and weight of the offspring (Zhou et al., 2008). Therefore, we chose a relatively mild 6% oxygen for hypoxic exposure (Wang et al., 2017). The HC and HE groups were treated with a gas mixture of 6% O_2_ and 94% N_2_ for 6 h a day, and both NH and exercise training were initiated with the flies between ages of 3 and 6 weeks old and were performed five times a week. Exercise training was performed from 930H to 1200H, and NH was provided from 1400H to 2000H. The NE, HC, and HE groups were all trained under standard environmental conditions as described above. The control group and the other three groups were collected and processed specifically to test their cardiac structure and function as well as behavior when these were 42 days old.

### Semi-Intact *Drosophila* Preparation and Cardiac Function

The semi-intact *Drosophila* hearts were prepared as follows. After the flies were anesthetized with CO_2_, the head was rapidly removed, then the ventral thorax and ventral abdominal cuticle were removed, exposing the abdomen. All internal organs except the heart and any abdominal fat were removed. Dissections were performed under oxygenated artificial *Drosophila* hemolymph (ADH) containing 108 mM NaCl_2_, 5 mM KCl, 2 mM CaCl_2_, 8 mM MgCl_2_, 1 mM NaH_2_PO_4_, 4 mM NaHCO_3_, 15 mM 4-(2-hydroxyethyl)-1-piperazinee thanesulfonic acid, 10 mM sucrose, and 5 mM trehalose, at pH 7.1 and room temperature (24°C) (Vogler and Ocorr, 2009). Then high-speed digital movies of beating hearts were taken with a Hamamatsu EMCCD 9300 camera (Hamamatsu, Inc.; 100 − 140 frames per second) at 120 − 140 fps for 30 s and were recorded with the HCImage software (Hamamatsu, Japan). The functional cardiac parameters of the four groups of flies were assessed using the semi-automatic optical heartbeat analysis software (SOHA, kindly gifted by Ocorr and Bodmer), which can accurately detect and quantify heart rate, heart period, arrhythmicity index, systolic and diastolic intervals, diastolic and systolic diameters, and percent fractional shortening. Moreover, M-modes, a qualitative record showing heart edge movement over time, were also produced using the optical heartbeat analysis program to further analyze the abnormal heart contractions (Fink et al., 2009). In addition, variations in the diameter of cardiac contraction and relaxation were calculated by subtracting the systolic diameter from the diastolic diameter.

### Transmission Electron Microscopy

After semi-intact *Drosophila* was prepared, heart tubes that were attached to the dorsal were pre-fixed in 2.5% glutaraldehyde buffered in PBS for 4 h at 4°C, then pre-embedded with 3% agar. After washing 3 × 10 min with 0. 1 mol⋅L^–1^ phosphate buffer, the hearts were post-fixed in 1% osmium tetroxide for 1 h at room temperature. Samples were subsequently dehydrated across an acetone gradient and then embedded in Embed-812 resin overnight. The following morning, the samples were polymerized at 60°C for 48 h, after which the fly heart tube was sectioned at 50−100 nm thickness and stained with 4% uranyl acetate and 0.25% lead citrate. Images were observed and saved with a HITACHI transmission electron microscope (HT-7700, Japan) operating at an accelerating voltage of 80−120 kV. Around four to five samples from each group were examined.

### Immunofluorescence Staining and Imaging

The semi-intact *Drosophila* hearts were prepared as previously described (Vogler and Ocorr, 2009). ADH was quickly replaced with a relaxing buffer (ADH that contains 10 mM EGTA) to ensure that all heart tubes were under a relaxed state. Then, the heart shape was fixed by replacing the relaxing buffer with fixative under room temperature condition for 20 min. The preparation was then washed thrice for 10 min with PBSTx, and trimmed specimens were incubated with rat monoclonal anti-myosin antibodies (MAC 147, 1:200, Abcam) diluted in PBSTx and incubated overnight at 4°C. The hearts were then washed thrice for 10 min with PBSTx at room temperature to remove the primary antibody and then incubated with labeled goat anti-rat IgG antibodies Alexa Fluor^®^ 647 (ab 150167, 1:500, Abcam) diluted in PBSTx for 1 h. The preparation was then rewashed thrice for 5 min with PBSTx and rinsed twice for 10 min with PBS to remove the Triton-X-100 and then mounted (Alayari et al., 2009). Immunofluorescence staining images were obtained with a confocal laser scanning microscope (LEICA TCS SP8). Around four to five samples from each group were evaluated.

### Western Blot Analysis

Sixty heart tubes were collected and homogenized in urea-based lysis buffer (Ngoka, 2008) with protease inhibitors at 4°C. The mixture was centrifuged at 12,000 rpm for 10 min at 4°C, and the supernatant was boiled for 10 min. SDS-PAGE and western blot analyses were performed following standard protocols (Chen et al., 2012). The primary antibodies used included rat monoclonal anti-myosin (MAC 147, 1:2,000, Abcam, Cambridge, MA, United States) and rat monoclonal anti-actin (MAC 237, 1:2000, Abcam, Cambridge, MA, United States), and the secondary antibody was goat anti-rat IgG H&L (HRP) (ab205720, 1:2000, Abcam, Cambridge, MA, United States). Densitometric analysis was performed by ImageJ software for quantitative assessment. Three technical repetitions in each group.

### Sleep–Activity Behavior Analysis

Sixteen flies from each group were separately transferred into 5 mm × 65 mm polycarbonate tubes with standard food at one side and a sponge on the other side. A12-h light/dark cycle (light turned on at 0600H, light turned off at 1800H; 0600H referred to zeitgeber time 0, and 1800H pertained to zeitgeber time 12) and a room temperature of 25°C were applied. The independent activities of each fly in 1-min bins were continuously monitored and recorded using the *Drosophila* Activity Monitoring System (DAMS) and Data Acquisition System (DAS, TriKinetics, Waltham, MA, United States). Locomotor activity monitoring lasts for at least 3 days, and the data for the first day were discarded due to the adjustment of flies to experimental monitor tube conditions (Linford et al., 2012; Chung et al., 2017).

The number of times the *Drosophila* moved back and forth in the tube to interrupt the intermediate infrared beam was considered as one activity count during the awake period. In this study, we analyzed hourly activity and total activity during the day and night, and the ratio of activity counts to waking time at daytime was represented as activity counts/waking time (AC/WT), which can reflect the health status of flies, i.e., a decrease in activity intensity indicates sickness or other impairment (Andretic and Shaw, 2005; Chiu et al., 2010). As previously described, *Drosophila* inactivity lasting for >5 min was defined as sleep (Hendricks et al., 2000; Shaw et al., 2000). The average total sleep, the average sleep bout duration and the sleep bout number were calculated based on the sleep definition (Andretic and Shaw, 2005; Liu et al., 2014). We further analyzed the number of first and second deep sleeps in fruit flies to quantify their sleep quality (van Alphen et al., 2013).

### Negative Geotaxis Behavior Assay

About 100 flies from each group were transferred into five 18-cm-long glass tubes with 2.8 cm in diameter, with ∼20 flies per tube, and the bottom of each tube was plugged with a centimeter-thick sponge to prevent escape and to avoid injury caused by falling. Flies were allowed to climb for 30 s and then were gently shaken to the bottom to induce the instinct of negative geotaxis. The flies’ whole complete climbing movement in tubes was videotaped. Adaptive climbing was performed in the first three times. The average climbing height of the flies in each vial was calculated by intercepting 15 s images of the fourth, fifth, and sixth climbing videos.

### Survival Analysis

No less than 100 virgin flies that were 42 days old were randomly selected from each group after hypoxia and/or exercise. The number of dead flies was recorded at 2200H each day. The number of surviving adults was compared to the original number of adults, and the survival rate each day was monitored. Differences in survival were analyzed by log-rank (Mantel–Cox) (Sujkowski et al., 2015).

### Statistical Analyses

The data were analyzed using SPSS version 16.0 software and presented as the mean ± the standard error of the mean (SEM). One-way ANOVA with least significant difference (LSD) tests was used to identify differences between the control group and the other three groups. Differences with *P* < 0.05 were considered statistically significant.

## Results

### Effects of Exercise and/or NH on Cardiac Function in Aged *Drosophila*

**FIGURE 1:**
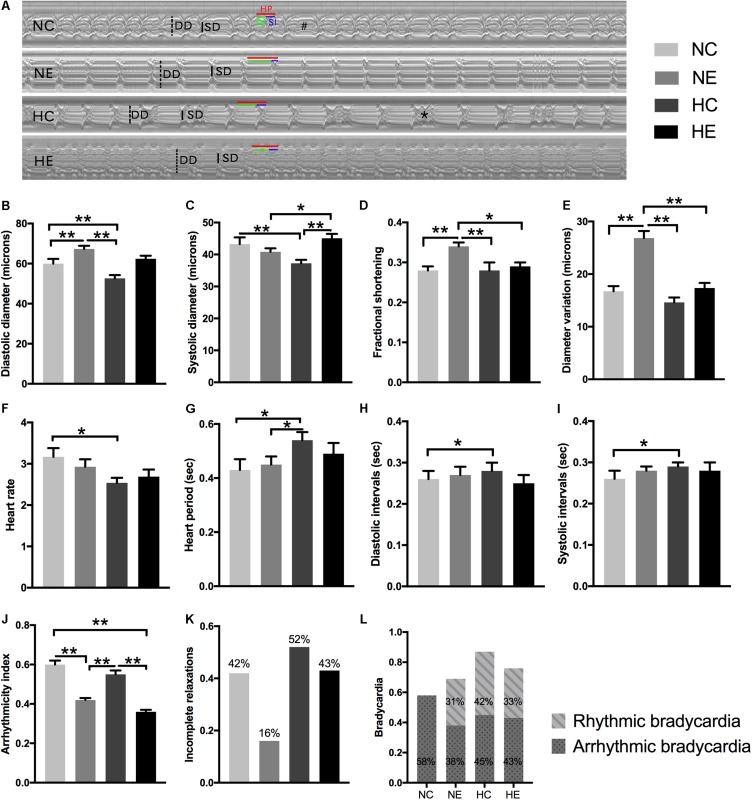
Effects of exercise and/or NH on cardiac function of elderly *Drosophila.* **(A)** Representative M-mode recordings (20 s time periods) in the second abdominal segment of the dissected heart from each group. Dotted and solid vertical lines in the M-mode traces indicate diastolic diameter (DD) and systolic diameter (SD) between the walls of the heart, respectively. Heart period (HP, horizontal red line), diastolic interval (DI, horizontal green line) and systolic interval (SI, horizontal blue line) were also marked. M-mode analysis revealed that bradycardia (indicated as “#”) and incomplete relaxations (shown as “^∗^”). **(B)** Diastolic diameter. **(C)** Systolic diameter. **(D)** Cardiac output, quantified as fractional shortening (FS) by calculating the percentage reduction in the diameter of the heart wall during contraction. **(E)** Diameter variation, subtract systolic diameter from diastolic diameter, reflect cardiac contractility, and diastolic compliance. **(F)** Heart rate (HR). **(G)** Heart period (HP). **(H)** Diastolic interval (DI). **(I)** Systolic interval (SI). **(J)** Arrhythmicity index (AI) calculated as the heart period standard deviation normalized to the median heart period. **(K,L)** evaluate the degree of abnormal cardiac contraction. **(K)** Incidence of incomplete relaxations, considered as very short DIs (<0.06 s) interrupt long SIs, correspond to the case of the “^∗^” mark in **(A)**. **(L)** Incidence of bradycardia, quantified as DIs longer than one second (approximately three times the length of the average DI), above is the incidence of rhythm bradycardia (during 30 s M-mode trace, every heartbeat has the same duration of DI and SI), and the following is the incidence of arrhythmic bradycardia (heartbeats occur unequal lengths of DI and SI), which corresponds to the case of the “#” mark in **(A)**. Data are displayed as mean ± SEM. Using one-way ANOVA with LSD tests among different groups, ^∗^indicates a *p*-value < 0.05, ^∗∗^indicates a *p*-value < 0.01. Sample size was 24–32 flies per group.

Cardiac aging is marked by a progressive decline in heart function, which contributes to abnormalities in diastolic relaxation, chamber filling, and/or passive myocardial stiffness (Nishimura et al., 2011). To study the alterations in *Drosophila* cardiac function induced by exercise and/or NH, we tested each group of flies with video-based cardiac performance assays as described above. Figure 1A is a representative M-mode trace of a heart tube from each group, showing the rhythmicity and dynamics of cardiac contraction and diastole. Hearts under separate exercise and hypoxic exposure showed different diameter variations (Figure 1E), which appear as significant enhancement of fractional shortening (Figure 1D) by improving the diastolic diameter (Figure 1B) after exercise training, thereby suggesting that exercise can delay the age-related decline of myocardial compliance and elasticity. However, NH dramatically diminished the heart lumen (Figures 1B,C) without affecting fractional shortening when compared to the control (Figure 1D). Furthermore, NH exposure resulted in prolongation of diastolic and systolic intervals (Figures 1H,I), ultimately leading to an increase in heart period (Figure 1G) and a decrease in heart rate (Figure 1F). Hypoxia combined with exercise did not show a better effect on cardiac systolic function, indicating that the hypoxia-exercise load did not delay cardiac aging.

Aging significantly influenced paroxysmal atrial fibrillation, leading to a more random time distribution (Yamashita et al., 1998). Similar to humans, the incidence of cardiac fibrillation dramatically increased in the control flies as these age (Ocorr et al., 2007), which is also well documented in our previous study (Zheng et al., 2017). To comprehensively analyze the effects of hypoxia and/or exercise on cardiac function, we further estimated the rhythmicity and abnormal heart contractions. Arrhythmicity index (AI) was calculated as the heart period standard deviation normalized to the median heart period, which is likely to be a more flexible and accurate method for generally quantifying arrhythmias (Fink et al., 2009); incomplete relaxations are considered as very short DIs (<0.06 s) that interrupt long SIs (representation of “^∗^” in Figure 1A); and bradycardia was quantified as DIs longer than one second (approximately three times the length of the average DI) (Fink et al., 2009) (representation of “#” in Figure 1A). To better distinguish benign and malignant bradycardia, we divided the bradycardia into rhythmic and arrhythmic bradycardia. The former indicates that during the 30 s M-mode trace, every heartbeat has the same duration of DI and SI. If one or more abnormalities occur, then these are considered arrhythmic bradycardias such as that depicted in the NC group in Figure 1A.

In our program, individual exercise can significantly improve cardiac arrhythmias and abnormal contractions, manifested as the incidence of AI (Figure 1J), incomplete relaxations (Figure 1K), and significantly reduced arrhythmic bradycardia (Figure 1L) and rhythmic bradycardia occurred, which also correspond to a reduction in heart rate. There was no significant improvement in the NH group except for the increase in rhythmic bradycardia (Figure 1L). The effect of hypoxia combined with exercise was similar to simple exercise in AI (Figure 1J) and bradycardia (Figure 1L), but did not reduce the occurrence of incomplete relaxation compared to the NC group (Figure 1K).

### Effects of Exercise and/or NH on Ultrastructure and Myofibrillar Components of Cardiomyocytes in Aged *Drosophila*

**FIGURE 2:**
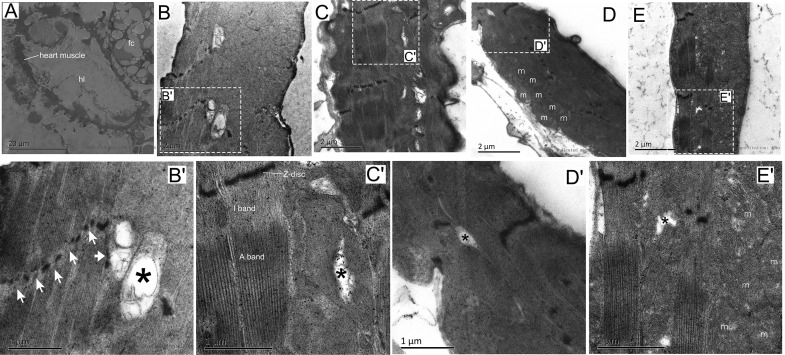
Effects of exercise and/or NH on the ultrastructure of *Drosophila* heart cells. **(A)** Cross ultra-thin sections through *Drosophila* cardiomyocytes located in abdominal segment 1. **(B,B′)** Distortion and defects in the Z-disk (white arrow in **B′**), fuzzy and degeneration myofibrillar (black asterisks in **B′**) were seen in aged flies using transmission electron microscopy (TEM). **(C,C′)** TEM micrographs through the contractile cardiomyocytes of exercise *Drosophila* showed clearly and continuously Z-disk, complete and well-arranged light band (I band) and dark band (A band). Note that small area myofibrillar degeneration was observed (black asterisk). **(D,D′)** Mitochondria in cardiomyocytes increased significantly after hypoxic exposure, while myofilaments were substantial blurred. Note that there is also minor myofibrillar degeneration (black asterisk). **(E,E′)** Hypoxia-exercise partially improved the arrangement of cardiac myofibrils, but the effect was not as good as a single exercise. Mitochondria were also observed after hypoxic-exercise. Myofibril degeneration is still observed (black asterisk). hl, heart lumen; m, mitochondria; fc, fat cell. Sample size of TEM was 4 – 5 independent samples per group.

To determine the effects of mild hypoxic exposure and/or modest exercise training in cardiac tissue at the ultrastructural level, we employed transmission electron microscopy (TEM) (Figure 2). TEM micrographs of a transverse section of contractile cardiomyocyte in the first abdominal segment were prepared according the structure of the *Drosophila* heart tube (Lehmacher et al., 2012). The cardiomyocytes form a lumen containing hemolymph (Figure 2A). Similar to changes in the aging fly muscle (Perkins and Tanentzapf, 2014), the heart muscle of the NC group also exhibited characteristic structural degradation, including Z-disk fragmentation (white arrow) and myofibril loss and ambiguity (black asterisks) (Figures 2B,B′). Compared to the control group, the NE group *Drosophila* showed distinct and intact sarcomeres with distinct I-bands and A-bands, and the myofilaments were arranged in a highly regular manner (Figures 2C,C′), which suggested that exercise can improve the structural degeneration of cardiac myocytes in senile *Drosophila*. Numerous mitochondria were observed in the heart cells of the NH flies, but myocardial filaments were more blurred than the controls (Figures 2D,D′). Hypoxia combined with exercise also showed a small amount of neatly arranged myofibrils. Mitochondria were also observed after hypoxia-exercise (Figures 2E,E′).

**FIGURE 3:**
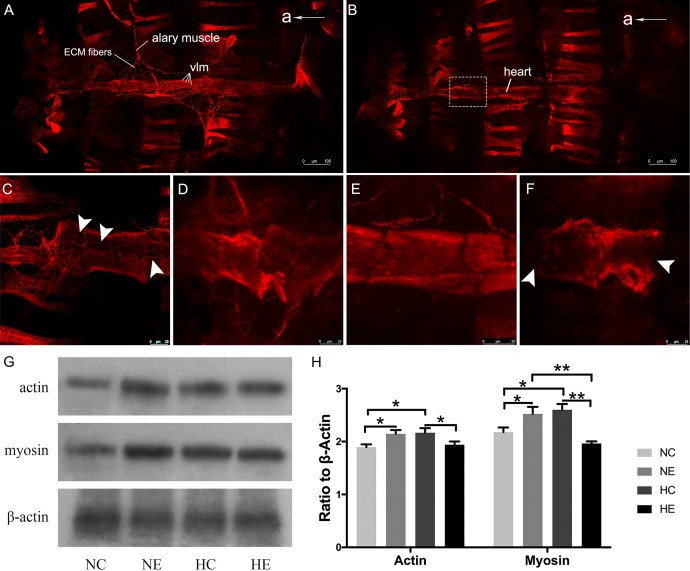
Effects of exercise and/or NH on myosin arrangements of cardiac contractile muscle layer and protein levels of myocardial myosin and actin. Representative immunofluorescence images of *Drosophila* heart stained with anti-myosin antibody as shown in **(A)** (ventral longitudinal muscle) and **(B)** (cardiac muscle) (at × 10 magnification). **(C–F)** Are magnified by centered on the area shown in the white dotted frame in **(B)**. **(C)** NC group; **(D)** NE group; **(E)** HC group; **(F)** HE group. The white arrows delineate the lack of patterned myosin immunoreactivity. Western blot analyses were performed with anti-actin, anti-myosin and anti-β-Actin antibodies. Representative blots are shown in **(G)**, the resulting bands were quantified and standardized to β-actin levels as **(H)**. a, anterior; vlm, ventral longitudinal muscle. Data are displayed as mean ± SEM. Using One-way ANOVA with LSD tests among different groups, ^∗^*P* < 0.05 and ^∗∗^*P* < 0.01. Sample size of immunofluorescence staining using four to five independent samples per group. Sample size of western blot analyses was 60 independent samples per group.

We further examined changes of myofibrillar components in the myocardium, including immunofluorescence of myosin and western blotting of myosin and actin. The *Drosophila* heart tube along the dorsal midline of the abdomen. After immunofluorescence staining, the contractile cardiomyocytes of the heart tube were detected by confocal laser scanning microscopy. Using different focal planes, we obtained fluorescence images of the myocardial contractile cells (Figure 3B) and ventral longitudinal muscles below the heart tube (Figure 3A). Myocardial cells from hearts showed a dense arrangement of myosin-containing myofibrils in both exercised and hypoxic flies (Figures 3D,E). However, Figures 3C,F show that the contractile myocardial cells of the control flies and files subjected to hypoxia-exercise exhibited a reduction in myosin content. Western blot analysis of the heart tubes showed that the NE and HC groups contained more myosin and actin than the control and hypoxia-exercise group (Figures 3G,H).

### Effects of Exercise and/or NH on Sleep-Activity Behavior of Aged *Drosophila*

**FIGURE 4:**
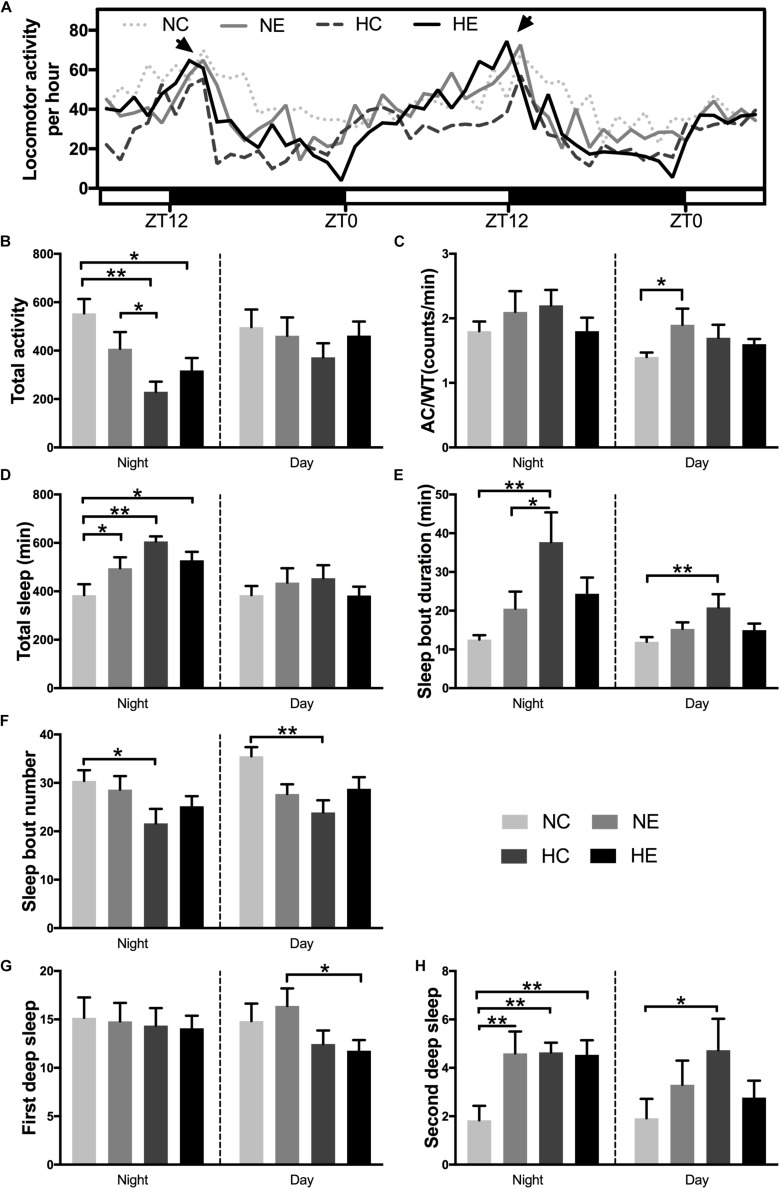
Effects of exercise and/or NH on sleep–activity behavior of aged *Drosophila.* Locomotor activity was recorded as the number of times a fly broke the path of an infrared beam at the midpoint of the tube. Periods of 5 min without beam crossing were regarded as a single period of sleep. **(A)** Averaged locomotor activity every hour of continuously 48 h for flies of each group. ZT, zeitgeber time. Light bar represents lights on, dark bar represents lights off. ZT0 and ZT12 represent the start and end of the photoperiod respectively. Arrows indicate “evening” peak. **(B)** Total locomotor activity at nighttime and daytime. **(C)** AC/WT, the ratio of activity counts to waking time at nighttime and daytime, indicate the activity intensity of *Drosophila*. **(D)** Total sleep at nighttime and daytime. **(E)** Sleep bout duration. **(F)** Sleep bout number. Panels **(G,H)** show the number of deep sleeps. **(G)** First deep sleep, the number of times sleep reaches 10 min. **(H)** Second deep sleep, the number of times sleep reaches 30 min. Data are displayed as mean ± SEM. Using one-way ANOVA with LSD tests among different groups, ^∗^ indicates a *p*-value < 0.05, ^∗∗^ indicates a *p*-value < 0.01. Sample size was 16 flies per group.

Under standard 12:12 LD conditions, wild-type *Drosophila* typically exhibit two peaks of activity; one centered around ZT0, designated as the “morning” peak, and another near ZT12, referred to as the “evening” peak (Chiu et al., 2010). A previous study has shown that circadian rhythm in sleep and behavior are significantly affected by aging (Kondratova and Kondratov, 2012). Similarly, in another study, we found that 6-week-old *Drosophila* had lower activity peaks both in the morning and evening compared to 3-week-old flies (Li et al., 2019). In this study, in *Drosophila* wild-type W^1118^ aged virgin females, after exposure to hypoxia and/or exercise for 3 weeks, a distinct “evening” peak was observed (Figure 4A, indicated by arrows), whereas the “morning” peak disappeared in the four groups of elderly flies. Hypoxia and/or exercise did not increase peak activity and instead reduced it to some extent (Figure 4A). We further analyzed the activity of the flies in the monitoring system (Figure 4A, ZT12-ZT12). After 3 weeks of hypoxia exposure, the total night activity of flies in the HC and HE groups decreased (Figure 4B), and the activity time also decreased synchronously (Supplementary Figure S1).

In addition to activity amount, we also evaluated the degree of locomotor activity. The average total number of counts per hour and overall night or day activities could not distinguish a poorly active but awake fly from a very active fly that sleeps for a large portion of a given hour. In contrast, activity counts/waking minute could distinguish hypoactive from hyperactive flies irrespective of sleep time. Moreover, counts/waking minute were a much better health indicator in flies, where a reduction in the intensity of activity is suggestive of a sick or otherwise impaired fly (Andretic and Shaw, 2005; Chiu et al., 2010). For example, flies that are simply sick may seem to sleep more because these are not as mobile. For these flies, their wake activity will be lower in relation to control animals (Chiu et al., 2010). In our study, exercise alone significantly increased daytime activity intensity in aged flies (Figure 4C). In addition, the application of hypoxia alone increased fly activity (Figure 4C).

One of the most consistent behavioral changes that occurs with age in humans is the loss of sleep consolidation, namely, increased daytime sleep and increased nighttime wakefulness (Koh et al., 2006). Meanwhile, a variety of causes, including nocturia, cause insomnia, more fragmented sleep, and reduced sleep quality in the elderly (Bliwise et al., 2009). The effects of aging in flies are almost identical to those in humans, i.e., older flies have more fragmented sleep, reduced total sleep, a lower arousal threshold, and fail to recover as much sleep after sleep deprivation (Vienne et al., 2016). Separate hypoxic exposure can significantly increase the total nighttime sleep (Figure 4D), prolong the sleep duration (Figure 4E) and reduce the sleep fragments (Figure 4F) of day and night in elderly flies. Exercise intervention (NE and HE group) only increased nighttime total sleep (Figure 4D), but had no significant effect on sleep bout duration and sleep bout number (Figures 4E,F).

One study has shown that responsiveness decreased gradually after the female flies began to sleep and reached a deeper sleep stage after ∼10 min of immobility, then responsiveness levels became lighter at around 20 min and later a second deeper sleep stage was observed at around 30 min (van Alphen et al., 2013). To better reflect the quality of sleep, we further analyzed two deep sleep episodes in aged *Drosophila*. Figures 4G,H are the number of times sleep reaches 10 and 30 min, corresponding to the duration of the first and second deep sleep, respectively. Either single exercise or hypoxia or the combination of both significantly increased the number of second deep sleep at night (Figure 4H), but had no effect on the first nighttime deep sleep. Although the total sleep in the HC group did not increase during the day, the quality of sleep significantly increased, as manifested by prolonged sleep duration (Figure 4E), decreased number of sleep bouts (Figure 4F), and increased number of second deep sleep (Figure 4H).

### Effects of Exercise and/or NH on Negative Geotaxis Behavior of Aged *Drosophila*

**FIGURE 5:**
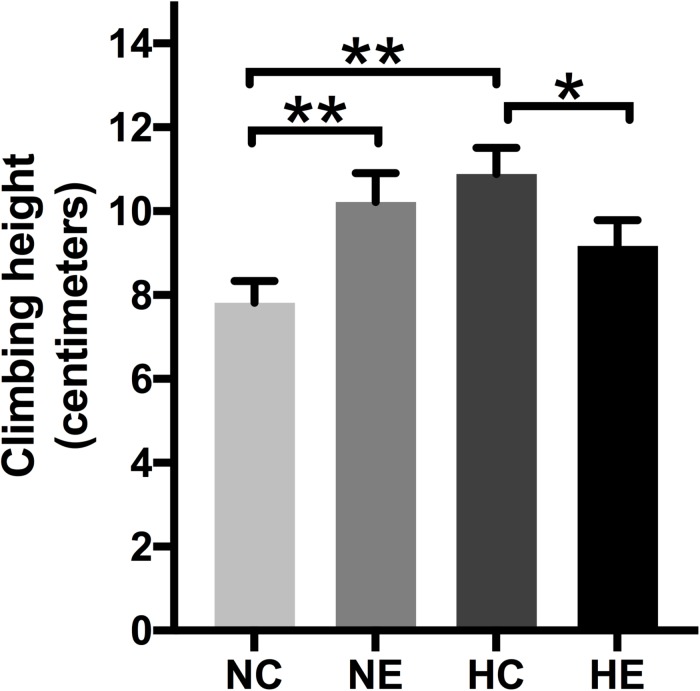
Effects of exercise and/or NH on climbing height of aged *Drosophila.* Climbing height induced by negative geotaxis for 15 s. One-way ANOVA followed by an LSD test among each group revealed that the distance climbed at 15 s increased after 3 weeks of exercise training and hypoxic exposure. Data are shown as mean ± SEM. ^∗^*P* < 0.05; ^∗∗^*P* < 0.01. Sample size was 90–100 flies per group.

The negative geotaxis of *Drosophila* is the ability to move vertically when frightened and declines with age, which is a commonly used to measure locomotion and senescence (Rhodenizer et al., 2008). Negative climbing height of 15 s was measured on the second day after the conclusion of exercise training and/or hypoxic exposure. The climbing height of the NE and HC groups was significantly higher than the controls (Figure 5), which suggests that implementing hypoxia or exercise alone can improve the negative geotaxis behavior of elderly *Drosophila*. The effect of hypoxia combined with exercise remained insignificant (Figure 5).

### Effects of Exercise and/or NH on the Lifespan and Mortality of Aged *Drosophila*

**FIGURE 6:**
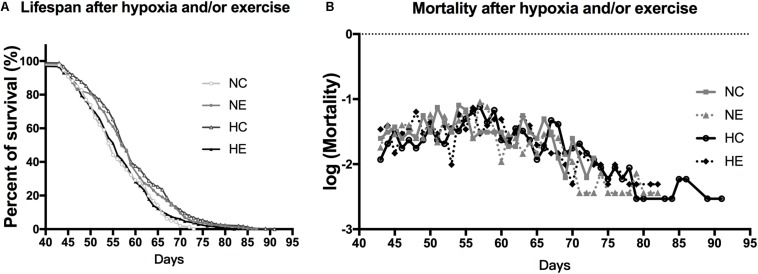
Effects of exercise and/or NH on lifespan of aged *Drosophila.* **(A)** Both exercise training and hypoxia exposure can prolong the lifespan of female flies (logrank; *p* < 0.001 and *p* < 0.000), while the combination of the two has no effect on longevity (logrank; *p* = 0.444). **(B)** Mortality after hypoxia and/or exercise is similar for all groups. The sample size of the NC, NE, HC, and HE groups were 167, 213, 302, and 294 flies, respectively.

Based on the finding of a previous study that the introduction of low amounts of leisure time physical activity to one’s daily routine may increase longevity (Moore et al., 2012) and older people living at the Tibetan Plateau tend to have a longer lifespan than those of living in lower altitudes (Li et al., 2017), we monitored the survival rate of flies after hypoxia and/or exercise intervention. The survival rate of the wild-type w^1118^ virgin female flies after implementing hypoxia or exercise alone showed a prolongation of the maximum. However, hypoxia combined with exercise showed minimal effects on life span. Mortality after hypoxia and/or exercise did not show major changes (Figure 6).

## Discussion

Heart-pumping function is determined mainly by the systolic function and the diastolic function and the percentage of reduction in the diameter of the heart wall during contraction. This reduction in diameter is referred to as the shortening fraction and can be used to estimate the cardiac contractility of the heart in *Drosophila* (Zheng et al., 2017). Similarly, analogous to observations in elderly humans, senescent hearts of *Drosophila* exhibit both impaired relaxation and increased myocardial stiffness, diastolic and systolic diameters were decreased, the systolic and diastolic intervals were prolonged, the cardiac output declined with age, and arrhythmias also shows a significant increase with age (Cammarato et al., 2008; Fink et al., 2009; Liao et al., 2014; Klassen et al., 2017). The basic contractile unit of myofibrils is called the sarcomere, which consists of antiparallel actin thin filaments and myosin thick filaments (Gonzalez-Morales et al., 2017). Loss of actin and myosin, which reflects the reduction of cardiac myofibrillar density, can lead to the reduction of cardiac contractile force (Cammarato et al., 2008) and several myocardial diseases (Tsoutsman et al., 2008). Many physiological and pathological conditions (Sivtseva et al., 2014), particularly aging, can result in systolic and diastolic dysfunction and ultimately evolve into heart failure (Borlaug and Redfield, 2011). For example, the cardiac aging of mammals is always accompanied by disordered arrangement and reduction of myocardial fibers (Amir et al., 2002). In the present investigation, aged *Drosophila* hearts possessed poorly organized Z-disks, fuzzy and degraded myofibrils (Figure 2), and the content of actin and myosin in the cardiomyocytes is also insufficient (Figure 3), suggesting that the structural and functional changes with age in cardiomyocytes of flies are remarkably similar to those in mammals.

Although at present there is no way to stop the biological aging process of organisms, previous studies (Leiser et al., 2013; Garatachea et al., 2015) have confirmed that mild hypoxic exposure and modest exercise training can delay functional decline. Just as Mahdiabadi et al. (2013) observed a significant increase in fractional shortening and end-systolic diameter with exercise. Similarly, our study also showed a statistical increase in fractional shortening in exercise training flies compared to the controls (Figure 1D), which is due to a significant increase in diastolic diameter (Figure 1B), thereby indicating that exercise induces a greater cardiac filling volume during diastole, and the resultant higher preload resulted in more cardiac output per beat, thereby enhancing the cardiac function of senile *Drosophila* (Figures 1B–D). These augmentations seem to be a benign physiological adaptation, which is different from dilated cardiomyopathy with large cardiac chamber accompanied by compromised fractional shortening (Pelliccia et al., 1999; Zheng et al., 2017). The increased fractional shortening and diastolic diameter during exercise in *Drosophila* are similar to the changes in cardiac function observed in humans after long-term endurance training and include an increase in left ventricular end diastolic diameter and end diastolic volume, left atrial as well as right atrial, and ventricular enlargement (Utomi et al., 2013; Santoro et al., 2014; Zheng et al., 2017). Does exercise training contribute to the increase in the cardiac systolic function of aged flies? As shown in Figure 1E, variations in the diameter of cardiac contraction and relaxation in the NE group were significantly greater than the controls, indicating that exercise training delayed the age-associated deterioration of cardiac contractility and diastolic compliance. Immunofluorescence staining assay and western blot analysis revealed that exercised flies displayed more extensive myosin-positive staining (Figure 3D) and the actin and myosin content of the heart tube also increased (Figures 3G,H), which show that exercise training can maintain the structural stabilization of thin and thick filaments. After intensive training, athletes showed a decrease in heart rate (Bonaduce et al., 1998), although 2.5 h of exercise in aged *Drosophila* exhibited a decrease in mean heart rate, although this was not statistically significant (Figure 1F), which is similar to the findings of our previous study (Zheng et al., 2017). Beneficially, our program significantly reduced the incidence of AI, incomplete relaxations, and arrhythmic bradycardia, and increased the incidence of rhythmic bradycardia (Figures 1J–L), which may be because exercise makes the arrangement of myofilament more regular (Figures 2C,C′). This asymptomatic bradycardia shows that the fruit fly has regular heartbeats, which is often seen in endurance athletes that do not present more arrhythmias than their non-bradycardic peers (Matelot et al., 2016).

Although the NH protocol implemented by our team was capable of significantly decreasing the percentage of elderly flies that exhibit heart failure under stress (Wang et al., 2017), it did not reveal any positive impact on the systolic and diastolic function of aged flies in the normoxic condition. More specifically, compared to the control group, M-mode records and quantitative analysis of elderly hypoxic exposure flies showed a significant decrease both in cardiac diastolic and systolic diameters (Figures 1A–C). Furthermore, *Drosophila* exposed to hypoxia had lower heart rates, which correspond to an increase in the incidence of bradycardia (Figure 1L), which seems to be an energy-saving activity, as they would be expected to reduce ATP demand in a low-oxygen environment (Zarndt et al., 2015). Hypoxia exposure does not reduce AI and incomplete relaxations as exercise training, but increases the rate of incomplete relaxation (Figures 1J,K), probably due to structural deterioration such as fuzzy myofibrils and incomplete sarcomeres (Figures 2D,D′). We also observed a positively stained myosin (Figure 3E), and an increase in myosin and actin protein content (Figures 3G,H) after hypoxic exposure. These reductions in cardiac function and changes in cardiac configuration and molecules may be due to a chronic cardiac stress response to a low-oxygen environment (Koolhaas et al., 2011; Zarndt et al., 2015) that resulted in cardiac remodeling due to long-term hypoxic exposure (Zarndt et al., 2015).

Compared to the NC group, hypoxia combined with exercise significantly reduced the incidence of AI (Figure 1J) and arrhythmic bradycardia and increased rhythmic bradycardia to some extent (Figure 1L), which may be reflects as a partial improvement in myofibril arrangement (Figures 2E,E′). However, there was no significant improvement in cardiac function and actin and myosin content after hypoxia combined with exercise, which indicated that the effect of simple superposition of hypoxia-exercise was not as good as that of hypoxic exercise alone.

One of the most consistent behavioral changes that occurs with age in humans is the loss of sleep consolidation, namely, increased daytime sleep and increased nighttime wakefulness in the elderly (Duffy and Czeisler, 2002; Pandi-Perumal et al., 2002; Koh et al., 2006). In fact, loss of sleep consolidation has been used as one of several measures of frailty in elderly people (Mitnitski et al., 2002). As a result, there is increasing awareness that treatment of their sleep problems could significantly improve the quality of life of older individuals (Koh et al., 2006). As a healthy, safe, inexpensive, and simple means, exercise can improve sleep quality, and offers a potentially attractive alternative or adjuvant treatment for insomnia (Youngstedt, 2005; Buman and King, 2010; Bonardi et al., 2016). As our results show, exercise increases night sleep time by reducing the nighttime activities and also increases the number of second deep sleep and daytime activity intensity (Figures 4B–D,H) and shows better sleep mode compared to the control group because individuals with better sleep quality exhibit higher activity during the day (Buman and King, 2010). Functional weakening of the circadian system with age has been observed in previous studies (Duffy and Czeisler, 2002; Kondratova and Kondratov, 2012). For example, the bimodal pattern activity weakens with age, which has been well documented in our previous studies (Zheng et al., 2017; Li et al., 2019) and is also consistent with the findings of other investigations (Koh et al., 2006; Vienne et al., 2016). However, exercise appears to be insensitive to improvement in circadian rest/activity rhythms (Figure 4A).

Does hypoxic or hypoxic combined exercise have a better effect? As stated in the Introduction, the results of previous research that evaluated the effects of altitude acclimatization on sleep quality are contradictory. The possible reasons for the contradiction may be the intensity of daytime activity (Hoshikawa et al., 2015). Under the same hypoxic conditions, exercise did not significantly increase the amount of sleep at night by reducing nighttime activities (Figures 4B,D) and both increased the number of deep sleeps at night (Figure 4H). Hypoxic exposure has the most significant effect on the sleep of elderly *Drosophila*, which not only increases the number of second deep sleep at night, but also reduces fragmentation, prolongs sleep duration, and the intensity of daytime activity also tends to increase. Hypoxia-exposed *Drosophila* showed improvements in daytime sleep efficiency without significantly increasing the total daytime sleep, i.e., sleep fragmentation was reduced, duration was extended, and the number of second deep sleep increased (Figures 4C,E,F,H). Combined with the hourly locomotor activity curve (Figure 4A), the activity level of *Drosophila* in the hypoxic group was low. This may be an energy-saving response after hypoxia, because sleep is a state that requires the least amount of energy expenditure, and energy savings can also be reduced activity-related energy expenditure (Penev, 2012; Spaeth et al., 2015).

*Drosophila* also exhibits several forms of locomotor behavior, including negative geotaxis, flying, and spontaneous walking, which decrease with age (Jones and Grotewiel, 2011). Exercise therapy is expected to improve skeletal muscle performance and maintain mobility in both humans and rodent models (Adams et al., 2008; Piazza et al., 2009). A recent study has shown that hypoxic exposure is an effective way to improve performance without exercising (Susta et al., 2017). Similarly, our study has shown that both exercise and NH could significantly improve the climbing ability of aged flies (Figure 5). In addition, the improvement of viability and the extension of maximum life span also showed the beneficial effect of exercise and hypoxia alone (Figure 6). However, the effect of hypoxia combined with exercise remains unclear. Some studies have shown that intermittent moderate hypoxic exposure with low-intensity exercise can promote aerobic exercise ability (Casas et al., 2000) or intermittent hypoxia-hyperoxia training and low-intensity exercise can promote the functional recovery of overtraining syndrome in athletes (Susta et al., 2017), suggesting that hypoxia combined with lower-intensity exercise may produce better results (Jones and Grotewiel, 2011), which may also guide our future research investigations on hypoxia-exercise.

## Conclusion

Exercise training and hypoxic exposure initiated later in life can increase the content of cardiac myosin and actin, improve the sleep quality, enhance activity ability and prolong life span in elderly *Drosophila*. In addition, exercise induced cardiac hypertrophy as well as cardiac function enhancement and ultrastructure of cardiomyocytes improvement, which seems to be a physiological adaptation to long-term physical exercise. In contrast, NH induced a decreased heart diameter and heart rate, an increase in the number of mitochondria, and deeper sleep, which may be an energy-saving response to long-term hypoxia. However, the effect of simple overlapping hypoxia and exercise is not obvious. Therefore, future investigations into the appropriate design of a combined hypoxia and exercise program aimed at delaying aging-related degenerative changes are warranted.

## Data Availability Statement

All datasets generated for this study are included in the article/Supplementary Material.

## Ethics Statement

This study was carried out in accordance with the recommendations of the Ethics Committee of Hunan Normal University. The protocol was approved by the Ethics Committee of Hunan Normal University.

## Author Contributions

HW and LZ conceived and designed the experiments. FY, HL, KL, and YL collected the samples. HW, QL, JL, and DC performed the experiments. QL and HW analyzed the data. QL, HW, and LZ wrote the manuscript. All authors have read and approved the final manuscript.

## Conflict of Interest

The authors declare that the research was conducted in the absence of any commercial or financial relationships that could be construed as a potential conflict of interest.
