# Supplementary material for: Effects of Modest Hypoxia and Exercise on Cardiac Function, Sleep-Activity, Negative Geotaxis Behavior of Aged Female Drosophila
Source: Front Physiol. 2020 Jan 21;10:1610. doi: 10.3389/fphys.2019.01610 (PMC6985434; doi:10.3389/fphys.2019.01610)
Supplement: Supplementary file 1 [file Image_1.pdf]

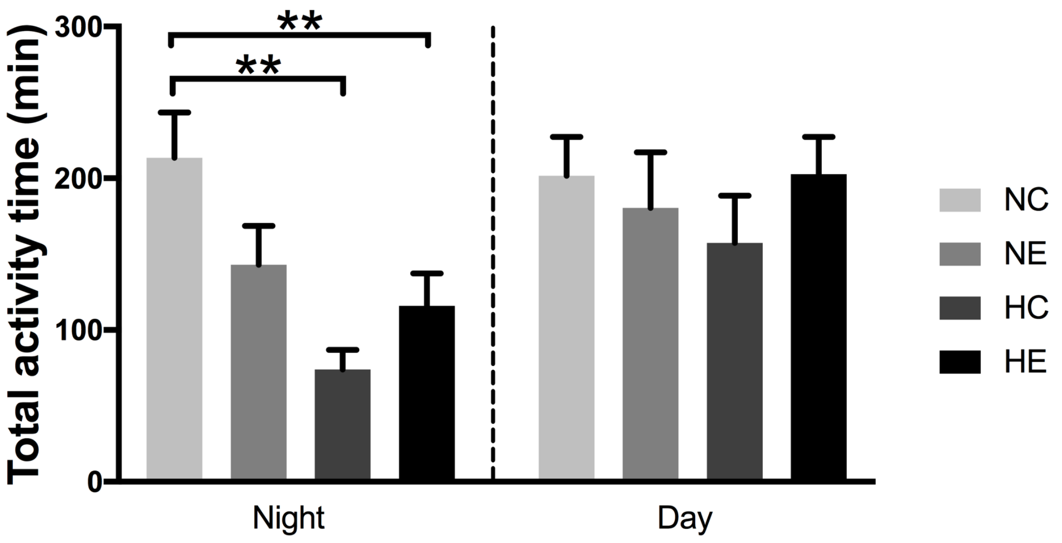

Fig. S1 Effects of exercise and/or NH on total activity time of aged *Drosophila*

Total activity time is the sum of the activity time in the awakening state. NH can reduce nocturnal activity time of aged female *Drosophila*. Data are displayed as mean ± SEM. Using one-way ANOVA with LSD tests among different groups, * indicates a p-value < 0.05, ** indicates a p-value < 0.01. Sample size was 16 flies per group.
